# Causal Associations of Smoking, Alcohol, Obesity, Sedentary Behavior, Hypertension, and Hyperglycemia With Retinal Vein Occlusion: A Mendelian Randomization Study

**DOI:** 10.2174/0113892029320896241218055907

**Published:** 2025-01-17

**Authors:** Danyi Li, Dong Liu, Yang Li, Zhongyan Lai, Wenjie Cao

**Affiliations:** 1 Department of Ophthalmology, Jiading District Central Hospital Affiliated Shanghai University of Medicine & Health Sciences, Shanghai, 201800, China

**Keywords:** Retinal vein occlusion, alcohol, hyperglycemia, smoking, mendelian randomization, hypertension

## Abstract

**Background:**

Retinal Vein Occlusion (RVO) is a common and main cause of blindness. Causal, possible risk variables must be identified to develop preventative strategies for RVO. Thus, we decided to evaluate whether smoking, alcohol, obesity, sedentary behaviour, hypertension, and hyperglycemia are associated with increased risk of RVO.

**Methods:**

The data sources of Mendelian Randomization (MR) study included FinnGen consortium and the original GWAS article. A total of 130,604 cases with RVO from FinnGen consortium and 12,136 cases with RVO from the original GWAS article. The exposures of this MR study included smoking, alcoholic consumption, obesity, sedentariness, hypertension, and hyperglycemia. The outcome of this MR study was RVO.

**Results:**

Genetic predispositions to alcohol consumption (OR (odds ratio), 1.124; 95%CI, 1.007-1.254; *P*=0.037) and hyperglycemia (OR, 1.108; 95%CI, 1.023-1.200; *P*=0.012) were associated with increased risks of RVO in FinnGen. There were no significant associations of genetically predicted consumption of smoking (OR, 1.037; 95%CI, 0.341-3.155; *P*=0.949), obesity (OR, 1.045; 95%CI, 0.975-1.119; *P*=0.213), sedentariness (OR, 1.022; 95%CI, 0.753-1.38-; *P*=0.888), or hypertension (OR, 0.944; 95%CI, 0.848-1.051; *P*=0.290) with RVO.

**Conclusion:**

This MR analysis provides genetic evidence that increased alcohol consumption and hyperglycemia may be causal risk factors for RVO. In addition, no genetic evidence in this MR analysis supported that there were causal associations between smoking, sedentariness, obesity and hypertension with RVO.

## 1 INTRODUCTION

Retinal Vein Occlusion (RVO) is a type of common diseases in the ophthalmological practice. Depending on the sites of occlusions, RVO can be divided into Branch Retinal Vein Occlusion (BRVO), Hemi-Retinal Vein Occlusion (HRVO), and Central Retinal Vein Occlusion (CRVO) [1]. RVO is commonly related with cardiovascular illnesses such as carotid stenosis and Takayasu arteritis. Compared with healthy people, patients with RVO are prone to suffer from macular edema, macular ischemia, optic nerve degeneration, vitreous hemorrhage, and vison loss *etc*. [2].

Exploring the pathogenic factors of RVO has never ceased before, and the acknowledged pathogenic factors of RVO include age, gender, hyperlipoidemia, cardiovascular diseases, inflammation, and diseases of the immune system [3]. Notably, both systemic and local inflammation have been reported to play an important role in the development of RVO [4-6]. Systemic inflammation can induce systemic hypercoagulability, and many inflammatory chemokines such as for tumor necrosis factor-α (TNF-α), interleukin-1β (IL-1β), and IL-6, are involved in the formation of thromboses. Local inflammation leads to the elevated levels of proinflammatory chemokines and decreased levels of anti-inflammatory chemokines, which also contributes to the development of RVO. In addition, it has been reported that epidermal growth factor homology domains (Ang/Tie) signaling pathway, Wnt signaling pathway, Phosphoinositide 3-kinase/Protein kinase B (PI3K/AKT) signaling pathway are also involved in the pathology and developments of RVO [7-9]. Thereby, research focusing on exploring the relationship between regulations of these signaling pathways and prognosis of RVO is urgently needed.

However, studies focusing on the causal associations of lifestyles (smoking, alcohol, obesity, and sedentariness) as well as metabolic risks (hypertension and hyperglycemia) with RVO are few and not credible enough. On one hand, many published studies are controversial. For example, Hashimoto Y *et al.* reported that high blood pressure and overweight contributed to the developments of RVO [10]. Still, Umeya R *et al.* reported that blood pressure and overweight were considered as confounders of the development of RVO [11]. On the other aspect, the majority of published researches are traditional retrospective studies, perspective studies, case reports, and reviews instead of randomized controlled trials (RCTs) [12, 13]. These types of studies have relatively low levels of evidence so that more convincing studies are urgently needed. However, RCTs may be not suitable for investigating and exploring the causes of RVO because there is a long lag duration between risky exposures and clinical features of RVO. In addition, RCTs are relatively time-consuming and high-priced to study the causal associations of lifestyles (smoking, alcohol, obesity, and sedentariness) as well as metabolic risks (hypertension and hyperglycemia) with RVO simultaneously. Therefore, it is significant for us to find a persuasive and effective approach to investigate the causal associations of common lifestyles (smoking, alcohol, obesity, and sedentariness) as well as metabolic risk factors (hypertension and hyperglycemia) with RVO.

Mendelian Randomization (MR) study is a new and promoting analysis method used to assess the causal associations between exposure factors as well as outcome variations. There is an important advantage of MR study that MR study is unlikely to be reversed by causality and confounders in that the two alleles (effect allele and other allele) of a Single Nucleotide Polymorphism (SNP) are randomly distributed by the Mendel’s laws [14]. Thus, we performed this MR analysis to assess the causal associations between common lifestyles (smoking, alcohol, obesity, and sedentariness), metabolic risk factors (hypertension as well as hyperglycemia), and RVO. Furthermore, we used summary statistical results from Genome Wide Association Study (GWAS) datasets.

Clarifying the causal associations between common lifestyles (smoking, alcohol, obesity, and sedentariness), metabolic risk factors (hypertension as well as hyperglycemia) and RVO is of a great significance to provide useful information about the preventions as well as treatments of RVO for the clinical work and the public.

## 2 MATERIALS AND METHODS

### 2.1 Genetic Instrument Selection

We explored the effects of common lifestyles (smoking, alcohol, obesity, and sedentariness), metabolic risk factors (hypertension as well as hyperglycemia). The framework of this MR analysis was illustrated in Fig. (**1**). Firstly, we chose SNPs for RVO that met the genome-wide significance criteria (*P*<5×10^-8^). But only a few significant SNPs of exposures or outcome were found using the *P*<5×10^-8^ threshold, so that the threshold was changed as *P*<5×10^-6^ for exposures and outcome [15]. Secondly, we removed SNPs which were in linkage disequilibrium (r^2^>0.01 and clump window<10,000 kb) and screened the remaining SNPs again. Finally, to ensure the vigor of the exposures, we performed the F statistic, and F statistic>10 was regarded as effectively robust to counteract the weak instrument bias.

The study has been evaluated by the Ethics Committee of Jiading District Central Hospital Affiliated Shanghai University of Medicine & Health Sciences and deemed not to require ethics approval.

### 2.2 Data Sources

#### 2.2.1 GWAS Data of Exposures

The smoking GWAS summary data were acquired from R10 version FinnGen study (https://storage.googleapis.com/finngen-public-data-r10/summary_stats/finngen_R10_SMOKING.gz), which included 3,155,619 samples (3,778 cases *vs* 151,841 controls) and 21,284,858 SNPs. The included population was European (Table **1**).

The alcohol GWAS summary data were acquired from R10 version FinnGen study (https://storage.googleapis.com/finngen-public-data-r10/summary_stats/finngen_ R10_AUD_SWEDISH.gz), which included 412,181 samples (24,070 cases *vs* 388,111 controls) and 21,306,346 SNPs. The included population was European (Table **1**).

The obesity GWAS summary data were acquired from R10 version FinnGen study (https://storage.googleapis.com/finngen-public-data-r10/summary_stats/finngen_R10_E4_ OBESITY.gz), which included 412,055 samples (23,971 cases *vs* 388,084 controls) and 21,306,347 SNPs. The included population was European (Table **1**).

The sedentariness GWAS summary data were acquired from original article [16], which included 400,364 samples (12,136 cases *vs* 388,228 controls) and 19,400,417 SNPs. The included population was European (Table **1**).

The hypertension GWAS summary data were acquired from R10 version FinnGen study (https://storage.googleapis.com/finngen-public-data-r10/summary_stats/finngen_ R10_O15_HYPTENSPREG.gz), which included 230,310 samples (16,417 cases *vs* 213,893 controls) and 21,298,922 SNPs. The included population was European (Table **1**).

The hyperglycemia GWAS summary data were acquired from R10 version FinnGen study (https://storage.googleapis.com/finngen-public-data-r10/summary_stats/finngen_ R10_KELA_DIAB_INSUL_ EXMORE.gz), which included 230,310 samples (62,368 cases *vs* 335,100 controls) and 21,306,094 SNPs. The included population was European (Table **1**).

#### 2.2.2 GWAS Data of RVO

RVO was diagnosed by International Classification of Diseases, 10th edition (ICD-10). The RVO GWAS summary data were acquired from R10 version FinnGen study (https://storage.googleapis.com/finngen-public-data-r10/summary_stats/finngen_R10_H7_RETIVASCOCCLU-SION.gz), which included 380,285 samples (3,635 cases *vs* 376,650 controls) and 21,305,778 SNPs. The included population was European.

### 2.3 MR Analysis

Inverse-variance weighted (IVW) method based on a multiplicative random-effects model was used as the main MR analytical strategy in this study [17]. IVW method was used to examine the possible causal associations between lifestyles (smoking, alcohol, obesity, and sedentariness), metabolic risk factors (hypertension and hyperglycemia) and RVO. Moreover, MR-egger, weighted median (WM), simple mode, and weighted mode were applied as supplementary MR analytical strategies in this study. Otherwise, we performed an MR-egger intercept analysis to evaluate the average pleiotropic effect [18]. To evaluate the robustness of the MR results, we applied the Cochran’s Q statistic to detect the heterogeneity. If the Q statistic<0.05, the heterogeneity was considered to exist [19]. In addition, the leave-one-out analysis was used to determine how eliminating one genetic variant from the MR analysis would influence the final results. We used RStudio-version 4.2.1 as the main software in this MR analysis and we also installed packages such as vroom and TwoSampleMR.

## 3 RESULTS

### 3.1 Association of Alcohol with the Risk of RVO

**Fig. (3):**
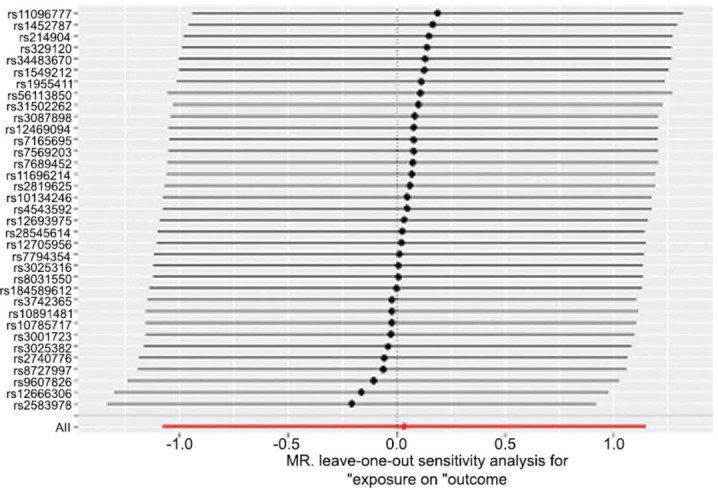
Leave-one-out plot: MR sensitivity analysis for alcohol consumption and RVO. MR, Mendelian randomization; RVO, retinal vein occlusion.

As depicted in Fig. (**2**) and Table **2**, alcohol consumption was associated with an increased risk of RVO (OR, 1.124; 95%CI, 1.007-1.254; *P*=0.037) analyzed by the IVW method. Furthermore, no heterogeneity was found among the instrumental SNPs effects (Q statistic=0.880). Moreover, the MR-egger intercept analysis (*p*=0.928) demonstrated that there was no average pleiotropy in this MR analysis. In addition, leave-one-out analysis indicated that the results of alcohol consumption and RVO were stable and convincing (Fig. **3**).

### 3.2 Association of Hyperglycemia with the Risk of RVO

**Fig. (5):**
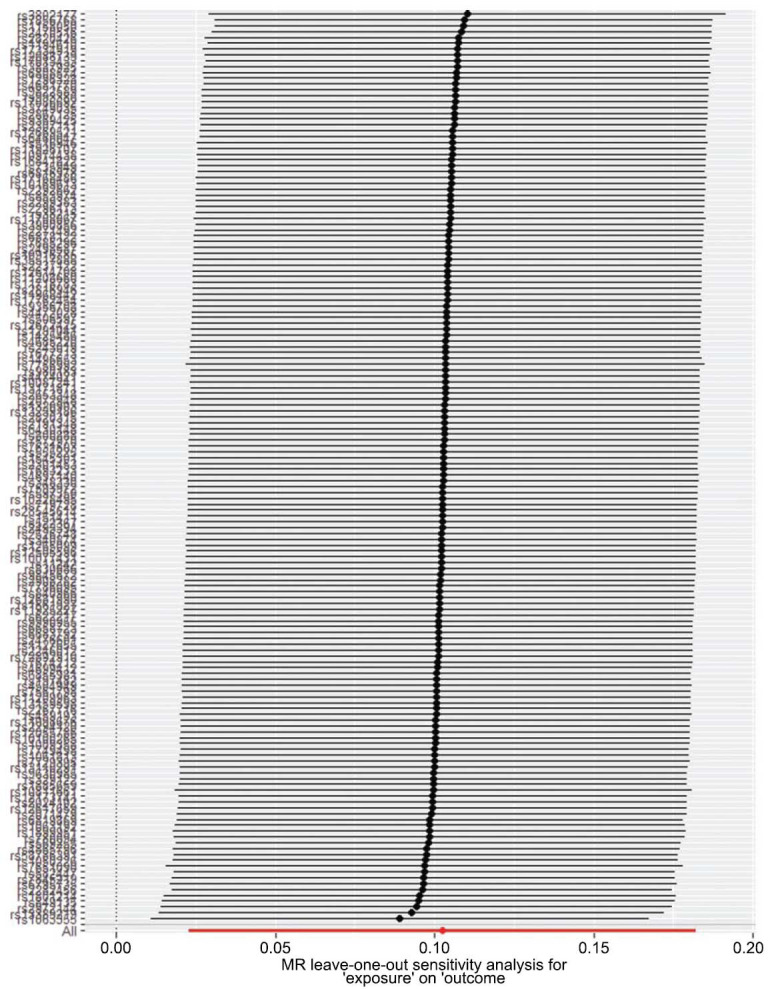
Leave-one-out plot: MR sensitivity analysis for hypertension and RVO. MR, Mendelian randomization; RVO, retinal vein occlusion.

As depicted in Fig. (**4**) and Table **2**, hyperglycemia was associated with an increased risk of RVO (OR, 1.108; 95%CI, 1.023-1.200; *P*=0.012) analyzed by the IVW method. Furthermore, no heterogeneity was found among the instrumental SNPs effects (Q statistic=0.235). Moreover, the MR-egger intercept analysis (*p*=0.222) demonstrated that there was no average pleiotropy in this MR analysis. In addition, leave-one-out analysis indicated that the results of hyperglycemia and RVO were stable and convincing (Fig. **5**).

### 3.3 Associations of Smoking, Obesity, Sedentariness, and Hypertension with the Risk of RVO

There was no evidence showing that smoking, obesity, sedentariness, as well as hypertension had causal associations with the risk of RVO analyzed by MR analytical strategies (Table **2**).

## 4 DISCUSSION

Our study applied an MR analysis to investigate potential causal associations between common lifestyles (smoking, alcohol, obesity, and sedentariness), metabolic risk factors (hypertension as well as hyperglycemia), and pathogenic risk of RVO. The genetic evidences supported that there was a potential causal association between alcohol consumption and advanced pathogenic risk of RVO, which was consistent with previous studies [20-23]. Furthermore, the cessation of alcohol consumption was associated with a decreased pathogenic risk of RVO compared with persistent alcohol consumption [24, 25]. In addition, the MR results also showed that there was a potential causal association between hyperglycemia and increased pathogenic risk of RVO. However, no sufficient evidenced supported that there were potential causal associations between smoking, obesity, sedentariness, as well as hypertension and RVO.

Traditional observational studies have consistently supported alcohol consumption as a risk factor for the development of RVO. For example, Chatzirallis A *et al.* reported that the life qualities of RVO patients were found to be affected by alcohol consumption [20]. Thapa R *et al.* showed that there was an increased risk of RVO among those patients who consumed alcohol [21]. Researchers also reported that the risk of CRVO decreased with increasing level of consuming alcohol [22]. Through applying this MR analysis, we also found that alcohol consumption was causally associated with pathogenic risk of RVO and the cessation of alcohol consumption played a protective role. The following mechanisms can be proposed to explain the association between alcohol consumption and development of RVO. Firstly, Oxidative stress have been confirmed to play a significant role in pathogenesis of RVO [26-30]. It has been known that alcohol consumption can generate free radicals by cytochrome CYP2E1, which not only damages but also causes the aggregation of the lens proteins, leading to the lens opacities. Subsequently, alcohol consumption can damage the retinal blood vessels, which causes increases in vascular permeability and changes in hemodynamics [31-33]. Finally, retinal atherosclerosis or vasoconstrictions can be induced by alcohol consumption, leading to the hypoxic and ischemic conditions in the retina, which causes the excessive expressions of vascular endothelial growth factor, thereby resulting in the pathogenesis and development of RVO [34-38]. Alcohol consumption can also cause vascular spasm and occlusion in the retina, thereby contributing to the pathogenesis of RVO and exacerbating the prognosis of RVO [39-41]. Furthermore, alcohol consumption may be associated with the release of inflammatory cytokines [42-44]. Interleukin (IL)-6 and IL-8 play significant roles of the pathogenesis of RVO, and these inflammatory cytokines are increased in RVO patients [45-48]. And alcohol consumption has been confirmed to induce the generations of IL-6 and IL-8, thus activating inflammatory reactions [6, 49].

Our finding also showed that hyperglycemia could increase pathogenic risk of RVO, which was consistent with previous studies [49-53]. As mentioned above, oxidative stress impairment was considered as an essential factor of the pathology of RVO. Chen W *et al.* found that persistent hyperglycemia could induce reactive oxygen species (ROS) generation and ROS could harm the retinal endothelial cells by damaging DNA, proteins, and lipids. Afterwards, the impaired hemodynamics of ROS-damaged retinal endothelial cells could lead to abnormal aggregation of platelets, which was a main cause of developing RVO [49, 54-56]. Moreover, not merely oxidative stress but also inflammation is responsible for the pathology of RVO. Beuse A *et al.* reported that exposure of hyperglycemia could significantly promote the secretion of IL-6 in Mueller glial cells, and IL-6 was associated with the increased pathogenic of RVO. Otherwise, hyperglycemia can lead to the glycation of the basement membranes of retinal veins and can cause it to thicken, which further affects the vascular permeability and blood flow, increasing the risk of the retinal ischemia [50, 57]. Furthermore, the occurrence of retinal ischemia can activate the generation of vascular endothelial growth factor (VEGF), which promotes angiogenesis [58]. However, these types of newly born vessels are unhealthy and prone to disruption, thereby greatly increasing the incidence rate of RVO and exacerbating its prognosis [59].

Interestingly, our findings about the causal associations between smoking as well as hypertension and RVO conflicted with previous studies and clinical experience [60-62]. Many studies have found that smoking and hypertension were risky factors of RVO [63-65]. However, in this MR study, we found that there was no causal association between smoking and RVO, and hypertension seemed to play a protective role in the pathogenesis of RVO (Table **2**). The mainly possible cause of this discrepancy was that there were differences between genetic levels and clinical manifestations.

As shown in Table **2**, although MR analysis did not support the causal association with smoking and RVO, the OR of the IVW method was 1.037, which supported smoking was the risk factor of RVO as well. The following mechanisms can be considered to explain the role played by smoking in the development of RVO. Smoking has been confirmed to reduce the levels of such antioxidants as heme oxygenase-1 (HO-1) and superoxide dismutase (SOD), which causes the disruption of the retinal pigment epithelium barrier and the formation of neovascularization [66-70]. Moreover, smoking can cause the retinal hypoxic condition, which can stimulate the generation of VEGF, leading to the retinal endothelial cell proliferation as well as neovascularization [71]. This mechanism is similar to the mechanism of alcohol consumption contributing to the pathogenesis of RVO. Furthermore, long duration of smoking can lead to the retinal vasospasm and stenosis, which also contributes to the pathogenesis of RVO [72]. In addition, the excessive inflammation induced by smoking is another important risky factor of RVO.

As for the results of hypertension and RVO, we found something conflicting and interesting. The conflicting thing we found was that there was no causal association between hypertension and RVO, which was different from commonly clinical experience. The interesting thing we found was that hypertension seemed to play a protective role in the pathogenesis of RVO, which was also different from previous studies [73-75]. Just like smoking and lung cancer, although lots of studies have reported that hypertension is a risk factor of RVO, no studies have confirmed that there was indeed a causal association between hypertension and RVO yet. The reasons why hypertension can play a protective role in RVO are listed as follows. Firstly, for elder patients, properly and slightly high blood pressure is helpful for maintaining the volume of retinal veins in order to avoid age-related retinal vasospasm and stenosis. Secondly, mild hypertension contributes to accelerate the flow rate of the blood, thereby alleviating the ischemic conditions of retinal veins, which can prevent RVO from deteriorating. Thirdly, mild hypertension can accelerate the removal of hazardous substances and inflammatory mediators, leading to a low incidence of inflammation and oxidative stress.

In this analysis, we did not find any associations between sedentariness and RVO. However, it is not clear whether sedentariness can contribute to increasing the risks of the pathogenesis of RVO or deteriorating its prognosis.

This MR analysis has several strengths. Firstly, this is the first MR analysis of exploring the casual associations between common lifestyles (smoking, alcohol, obesity, and sedentariness) as well as common metabolic risks (hypertension and hyperglycemia) and RVO. Using this type of emerging analysis method is important for us to delve deeply into the pathogenesis and treatments of RVO. Secondly, the application of MR analysis can avoid reverse causation bias and confounding, which has an advantage over traditional observational studies. Thirdly, this MR analysis can enhance the statistical power in that it depends on the large GWAS summary data for both exposure factors and outcome. Applying GWAS summary data can make the research more objective and convincing than traditional observational studies. Finally, in this MR analysis, we also performed MR-egger intercept analysis, sensitivity analyses, Cochran’s Q statistic, as well as leave-one-out analysis to detect the average pleiotropic effect, the robustness and heterogeneity of the MR results.

However, this MR analysis also has some limitations. The population included in this study is European, so the conclusions drawn from this study may be not suitable for Asian and African. In addition, MR analysis can only explore the linear relationship between exposure factors and outcomes, instead of analyzing the non-linear relationships between exposure factors and outcomes.

## CONCLUSION

**Fig. (6):**
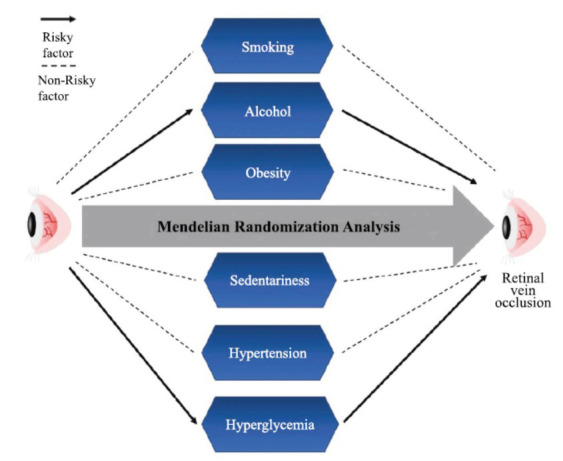
Conclusion figure of this MR analysis. Alcohol consumption and hyperglycemia have potential causal associations with the increased pathological risk of RVO. Smoking, obesity, sedentariness, and hypertension were not associated with the increased pathological risk of RVO.

We reported the genetic evidence that alcohol consumption and hyperglycemia have potential causal associations with the increased pathological risk of RVO. However, we found no genetic evidence that smoking, obesity, sedentariness, and hypertension were associated with the increased pathological risk of RVO (Fig. **6**). To reduce the prevalence of RVO, the ophthalmologists should appeal for alcohol consumption cessation and urgent treatments of hyperglycemia.

## Figures and Tables

**Fig. (1):**
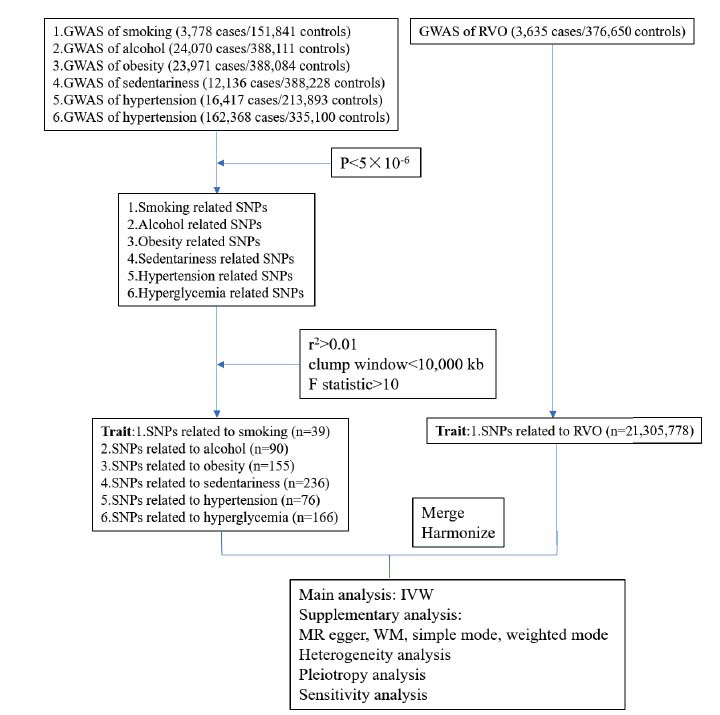
Framework of this MR analysis. RVO, retinal vein occlusion. SNPs, single nuclear polymorphisms; GWAS, genome-wide association studies; IVW analysis, inverse-variance weighted analysis; MW analysis, median weight analysis.

**Fig. (2):**
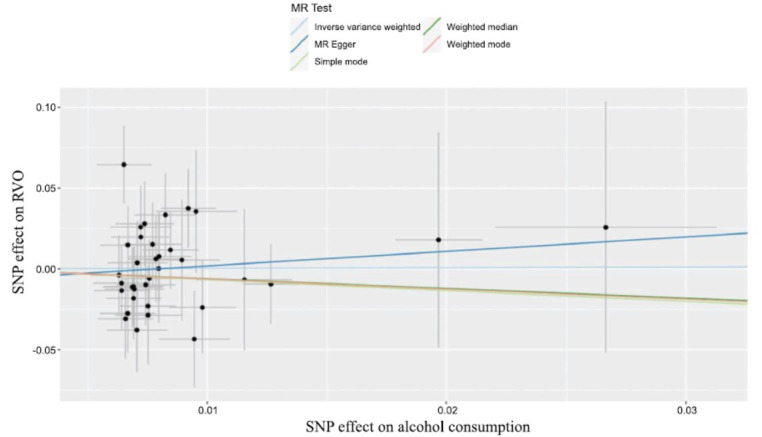
Scatter plot of the SNP effects on alcohol consumption and RVO, with the slope of each line corresponding to the estimated MR effect per method. RVO, retinal vein occlusion; OR, odds ratio; CI, confidence interval; MR, Mendelian randomization; SNPs, single nucleotide polymorphisms.

**Fig. (4):**
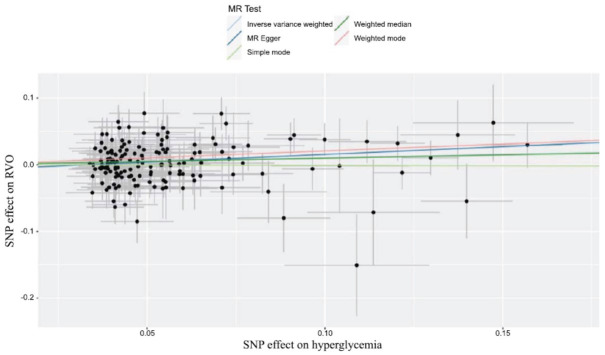
Scatter plot of the SNP effects on hyperglycemia and RVO, with the slope of each line corresponding to the estimated MR effect per method. RVO, retinal vein occlusion; OR, odds ratio; CI, confidence interval; MR, Mendelian randomization; SNPs, single nucleotide polymorphisms.

**Table 1:** Characteristics of GWAS consortiums used for each exposure.

**Exposures**	**Sources**	**Sample Size (cases/controls)**	**nSNPs**	**Population**
Smoking	FinnGen study	3,778/151,841	21,284,858	European
Alcohol	FinnGen study	24,070/388,111	21,306,346	European
Obesity	FinnGen study	23,971/388,084	21,306,347	European
Sedentariness	Original GWAS article	12,136/388,228	19,400,417	European
Hypertension	FinnGen study	16,417/213,893	21,298,922	European
Hyperglycemia	FinnGen study	62,368/335,100	21,306,094	European

**Table 2:** Associations of genetic predispositions to smoking, alcohol, obesity, sedentariness, hypertension, as well as hyperglycemia with RVO in MR analytical strategies.

**Exposures**	**nSNPs**	**OR (95% CI)**	**Beta (SE)**	**P**	**Q Statistic**	**MR-Egger Intercept (p)**
Smoking	-	-	-	-	-	-
IVW	35	1.037 (0.341-3.155)	0.568	0.949	0.761	0.708
MR Egger	35	2.453 (0.025-243.774)	0.822	0.705	-	-
WM	35	0.547 (0.109-2.737)	0.822	0.463	-	-
Simple mode	35	0.512 (0.022-11.895)	1.604	0.680	-	-
Weighted mode	35	0.536 (0.027-10.690)	1.527	0.685	-	-
Alcohol	-	-	-	-	-	-
IVW	73	1.124 (1.007-1.254)	0.056	0.037	0.880	0.928
MR Egger	73	1.137 (0.855-1.513)	0.146	0.380	-	-
WM	73	1.181 (1.007-1.386)	0.081	0.041	-	-
Simple mode	73	1.283 (0.847-1.944)	0.212	0.244	-	-
Weighted mode	73	1.275 (0.908-1.789)	0.173	0.164	-	-
Obesity	-	-	-	-	-	-
IVW	181	1.048 (0.975-1.119)	0.035	0.213	0.267	0.771
MR Egger	181	1.017 (0.836-1.236)	0.100	0.868	-	-
WM	181	1.081 (0.963-1.214)	0.059	0.187	-	-
Simple mode	181	1.096 (0.813-1.476)	0.152	0.549	-	-
Weighted mode	181	1.088 (0.896-1.321)	0.099	0.394	-	-
Sedentariness	-	-	-	-	-	-
IVW	190	1.022 (0.753-1.389)	0.156	0.888	0.224	0.553
MR Egger	190	0.713 (0.209-2.430)	0.626	0.590	-	-
WM	190	1.234 (0.798-1.912)	0.223	0.344	-	-
Simple mode	190	1.856 (0.512-6.723)	0.627	0.348	-	-
Weighted mode	190	1.636 (0.468-5.717)	0.638	0.441	-	-
Hypertension	-	-	-	-	-	-
IVW	68	0.944 (0.848-1.051)	0.055	0.290	0.0464	0.178
MR Egger	68	0.753 (0.534-1.060)	0.175	0.109	-	-
WM	68	0.871 (0.752-1.009)	0.075	0.066	-	-
Simple mode	68	0.740 (0.537-1.018)	0.163	0.068	-	-
Weighted mode	68	0.764 (0.580-1.007)	0.141	0.061	-	-
Hyperglycemia	-	-	-	-	-	-
IVW	156	1.108 (1.023-1.200)	0.041	0.012	0.235	0.222
MR Egger	156	1.260 (1.011-1.572)	0.113	0.042	-	-
WM	156	1.103 (0.977-1.247)	0.062	0.114	-	-
Simple mode	156	0.991 (0.728-1.347)	0.157	0.952	-	-
Weighted mode	156	1.230 (0.988-1.530)	0.111	0.065	-	-

## Data Availability

The authors confirm that the data supporting the findings of this research are available within the article.

## LIST OF ABBREVIATIONS

BRVO: Branch Retinal Vein Occlusion
CRVO: Central Retinal Vein Occlusion
GWAS: Genome Wide Association Study
HRVO: Hemi-Retinal Vein Occlusion
IL-1β: Interleukin-1β
IVW: Inverse-variance Weighted
MR: Mendelian Randomization
RCTs: Randomized Controlled Trials
ROS: Reactive Oxygen Species
RVO: Retinal Vein Occlusion
SNP: Single Nucleotide Polymorphism
TNF-α: Tumor Necrosis Factor-α
VEGF: Vascular Endothelial Growth Factor
